# Measurement tools to assess activities of daily living in patients with Parkinson’s disease: A systematic review

**DOI:** 10.3389/fnins.2022.945398

**Published:** 2022-07-20

**Authors:** Raquel Bouça-Machado, Adriana Fernandes, Carlo Ranzato, Duane Beneby, Hipólito Nzwalo, Joaquim J. Ferreira

**Affiliations:** ^1^Instituto de Medicina Molecular João Lobo Antunes, Faculdade de Medicina, Universidade de Lisboa, Lisbon, Portugal; ^2^CNS–Campus Neuroloígico, Torres Vedras, Portugal; ^3^Faculty of Medicine and Biomedical Sciences, University of Algarve, Faro, Portugal; ^4^European School of Physiotherapy, Hogeschool Van Amsterdam, Amsterdam, Netherlands; ^5^Algarve Biomedical Center Research Institute, Algarve, Portugal; ^6^Laboratory of Clinical Pharmacology and Therapeutics, Faculdade de Medicina, Universidade de Lisboa, Lisbon, Portugal

**Keywords:** Movement disorders, activities of daily living, ADL, measurement tools, clinical scales, outcome measures, patients reported outcomes, assessment

## Abstract

**Introduction:**

Parkinson’s disease (PD) is associated with a progressive inability to accomplish essential activities of daily living (ADL) resulting in a loss of autonomy and quality of life. Accurate measurement of ADL in PD is important to monitor disease progression and optimize care. Despite its relevance, it is still unclear which measurement instruments are the most suitable for evaluating ADL in people with PD.

**Objective:**

To identify and critically appraise which measurement instruments have been used to assess ADL in PD.

**Methods:**

A systematic review was conducted using the databases CENTRAL, MEDLINE, and PEDro from their inception to October 2021 to identify all observational and experimental studies conducted in PD or atypical parkinsonism that included an ADL assessment. Titles and abstracts were screened independently by two authors. The clinimetric properties of the measurement instruments were assessed, and the instruments were classified as “recommended,” “suggested,” or “listed.”

**Results:**

A total of 129 articles were included, with 37 measurement instruments used. The Unified Parkinson’s Disease Rating Scale (UPDRS), the Schwab & England ADL scale (S&E scale), the Movement Disorder Society Unified Parkinson’s Disease Rating Scale (MDS-UPDRS), the Barthel Index, the Lawton-Brody Instrumental Activities of Daily Living Scale, the Functional Independence Measure (FIM) and the Alzheimer’s Disease Cooperative Study – ADL (ADCS-ADL) scale were the seven most frequently cited measurement instruments. Of these, only two included an assessment of basic and instrumental ADL.

**Conclusion:**

MDS-UPDRS and the S&E scale were the only two scales that could be classified as recommended. For the MDS-UPDRS, either the full version or only Part II, which is focused on ADL, can be used. Future studies should explore the use of wearable devices to assess ADL remotely and more continuously.

## Introduction

Parkinson’s disease (PD) is a heterogeneous neurodegenerative disease characterized by a wide range of motor and non-motor symptoms (Bloem et al., 2021). Regardless of the therapeutic options available, disease progression usually leads to impaired functionality, loss of autonomy, and increasing dependence (Lee et al., 2016).

The term “Activities of Daily Living” (ADL) refers to the fundamental skills required for self-care, such as eating, bathing, and mobility (Katz, 1983). There are two types of ADL: basic (also known as physical) and instrumental. Personal hygiene or grooming, dressing, toileting, transferring or ambulating, and eating are all basic ADLs. More complex activities related to being able to live independently in the community, such as financial planning and medication management, food preparation, housekeeping, and communication with others (telephone, email), are included in the instrumental ADL (Edemekong et al., 2022). ADLs are a measure of a person’s functional status and are dependent on the motor, cognitive, and perceptual abilities. Patients’ inability to perform ADL leads to reliance on others and/or mechanical devices, resulting in unsafe conditions and poor quality of life (De Vriendt et al., 2012). It also contributes to caregivers’ burden, a multifaceted strain endured by a person who cares for an ill, which is associated with a lower quality of life, physical and psychological health problems of the caregiver, and decreased care provision to the patient (Liu et al., 2020).

As PD progresses, patients’ ability to perform ADL deteriorates (Schenkman et al., 2002; Hariz and Forsgren, 2011). Evaluating ADL limitations can aid in disease progression monitoring, care optimization, and disease burden reduction (Bloem et al., 2021). Standardized outcome measures are recommended to facilitate effective communication between healthcare professionals, allow for discussion with patients and families about the course of the disease and treatment plan, and compare clinical study results, all of which contribute to better patient care (Foster, 2014). Even though ADL is a relevant topic and a common outcome in PD clinical studies, the best measurement instruments for assessing ADL in PD patients are unknown (Lee et al., 2016). As a result, we conducted a systematic review of the published literature to identify and critically evaluate the measurement instruments used to assess ADL in PD. Recommendations were made based on the results.

## Methods

### Literature search

We searched CENTRAL, MEDLINE, and PEDro from their inception to October 2021 using the following keywords “Parkinson*,” “Activity* daily living,” and “ADL.” Reference lists from the identified articles were cross-checked to identify any further potentially eligible studies.

### Study selection

Observational and experimental clinical studies conducted in PD and/or atypical parkinsonism were included. Studies had to include an ADL assessment and describe the measurement tool used. Studies written in languages other than English, Portuguese, and Spanish were excluded. Two authors independently screened abstracts obtained from the database search. The full texts of potentially relevant articles were retrieved for further assessment. Disagreements were resolved by consensus or by consultation with a third reviewer.

### Data extraction

The following data from the individual studies were included in a pre-piloted form: general information (authors, journal and year of publication, study design, population and sample size, intervention and control conditions, and primary outcome); ADL measurement tools (name of measurement instruments, type of instrument, and timings of measurement) and measurement instrument classification.

### Assessment of measurement instruments

Based on criteria used in previous reviews, measurement instruments were classified as recommended, suggested, or listed (Antonini et al., 2011; Elble et al., 2013). These included: being developed and used in PD patients (A), being used in published studies by people other than the developers (B), and “successful” clinimetric testing (C). Measurement instruments were classified as recommended if all three criteria were met; suggested if two of the criteria were met; and listed if only one criterion was met.

The search for studies evaluating the clinimetric properties of the included measurement tools was based on previous research (Terwee et al., 2012; Bloem et al., 2016) and the references provided for each measurement tool in the included studies.

### Statistical analysis

The primary outcome was to identify the measurement instruments currently used to evaluate ADL in people with PD. Descriptive data were summarized using frequencies and percentages.

## Results

### General data

**FIGURE 1:**
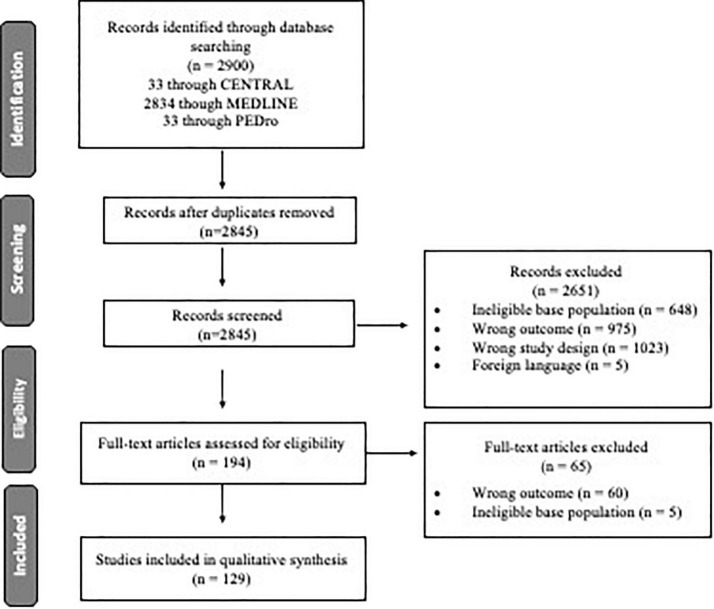
Flow diagram of the study selection process.

The electronic searches identified 2,900 citations (Figure 1). A total of 415 full-text articles were assessed for eligibility and 129 were included in the review. Wrong outcome (*n* = 2167), wrong study design (*n* = 973), and ineligible base population (*n* = 653) were the most common reasons for exclusion.

**FIGURE 2:**
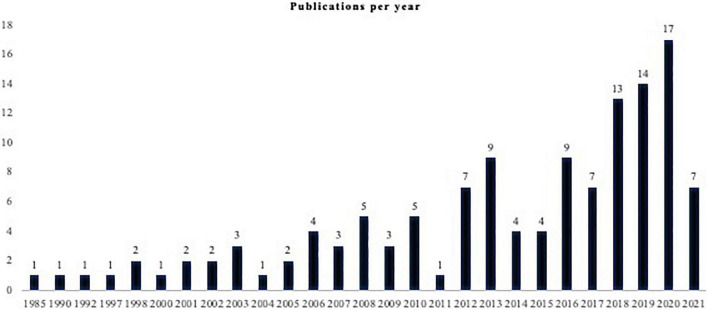
Publications per year.

Over time, the number of studies evaluating PD has increased (Figure 2). Of the 129 included studies, the three most common study designs were cross-sectional (31%, *n* = 40), prospective cohort (27.1%, *n* = 35) and randomized controlled studies (19.4%, *n* = 25). The median sample size was 64 (7, 3,777) patients, with a study duration ranging from one to 180 months. ADL was the primary outcome in 88.4% (*n* = 114) of the studies.

### Measurement instruments

A total of 37 measurement instruments were used in the included 129 studies. Of these, 46% (*n* = 17) can be used as patient- or clinician-reported outcomes, 27.2% (*n* = 10) were clinician-reported outcomes (ClinRO), 16.2% (*n* = 6) observer-reported outcomes (ObsRO), and 8.1% (*n* = 3) were patient-reported outcomes (PRO). Wearable sensors were used in one of the studies to evaluate specific movements related to ADL (sitting down on a chair, standing up from a chair, reaching, walking, and running) (Nguyen et al., 2018).

The most frequently cited measurement instruments (used in at least five studies) are summarized in Table 1, with a more detailed description below.

**TABLE 1:** Characteristics and classification of the most cited measurement tools.

Instrument	Types of assessment	Type of ADL	Developed for use in PD	Scale has been applied to PD populations	Used by other groups beyond the original developing group	Clinimetric properties studied for PD population	Recommendation level
MDS-UPDRS	ClinRO	Basic and instrumental ADL	Yes	Yes	Yes	Internal consistency, concurrent validity, face validity. Correlation of Part II with disability measures and quality of life scales.	Recommended
S&E ADL	ClinRO | PRO	Not specify	Yes	Yes	Yes	Test-retest reliability, inter-rater/intra-rater reliability, minimal detectable changes, and minimal clinical important difference	Recommended
Barthel ADL	ClinRO | PRO	Basic ADL	No	Yes	Yes	Test-retest reliability, inter-rater/intra-rater reliability, internal consistency, convergent validity	Suggested
Lawton-Brody	ClinRO | PRO	Instrumental ADL	No	Yes	Yes	No	Listed
FIM	ClinRO | ObsRO	Basic ADL	No	Yes	Yes	No	Listed
ADCS-ADL	ClinRO	Basic and instrumental ADL	No	Yes	Yes	No	Listed
UPDRS	ClinRO	Basic and instrumental ADL	Yes	Yes	Yes	Test–retest reliability (all and ADL section), inter-rater reliability, internal consistency, criterion and convergent validity. Regarding Part II, floor and ceiling effects, convergent validity, reliability, and standard error of measurement were found to be adequate.	Recommended, but replaced by the MDS-UPDRS

Regarding the Unified Parkinson’s Disease Rating Scale (UPDRS) and the Movement Disorder Society Unified Parkinson’s Disease Rating Scale (MDS-UPDRS), the included studies only used the section dedicated to ADL (Part II). We chose to present the scale in its entirety in this review, stating whether Part II can be used independently and what its benefits are.

#### Unified Parkinson’s disease rating scale

Unified Parkinson’s Disease Rating Scale Part II was used in 34.1% (*n* = 44) of the included studies.

*Construct assessed:* Disease severity.

*Test description:* The UPDRS is a disease-specific rating scale containing 55 items grouped into four subscales: Part I – mentation, behavior, and mood; Part II – activities of daily living; Part III – motor exam; and Part IV – complications of therapy (in the past week). A lower score represents less disability in performing ADL in PD. According to its description, the average time required to administer the full scale is between 10 and 20 min (Movement Disorder Society Task Force on Rating Scales for Parkinson’s Disease, 2003).

*Clinimetric properties:* Specifically designed for the PD population. Test-retest reliability is excellent (ICC = 0.92) for the entire scale and substantial (ICC = 0.85) for the ADL section. It has adequate internal consistency (Cronbach’s alpha = 0.96) and inter-rater reliability (*k* ≥ 0.4), criterion, and convergent validity. The clinimetric properties for Part II as an independent scale were already studied and showed that floor and ceiling effects, convergent validity, reliability, and standard error of measurement, found to be adequate (Martinez-Martin et al., 2006), suggesting that the UPDRS Part II generally would be considered a psychometrically sound tool for measuring ADL outcomes in people with PD (Ramaker et al., 2002; Siderowf et al., 2002; Hariz et al., 2003; Martínez-Martín et al., 2003; Hagell, 2019).

*Feasibility:* The UPDRS has several advantages, including the ability to provide a comprehensive assessment of the main features of PD while requiring little material (just a pencil or a pen and a chair). Its main drawbacks are its reliance on the expertise of the evaluator and the amount of time it takes. Although it is estimated to take 10–20 min, due to the numerous topics that must be discussed with the patient, it may take longer, limiting its use in clinical practice. The Movement Disorder Society (MDS) sponsored a critique of the UPDRS in 2001, which praised the scale’s strengths while pointing out several ambiguities, flaws, and areas that needed to be included to reflect current scientific developments. In 2008, the MDS developed a new version of the scale, which addressed some of the original scale’s flaws and included several clinically relevant PD-related issues that were underrepresented in the original version. Although the new version of the scale was recommended, the transition was not immediate as the old version had been used in several studies after 2008 (Hoehn and Yahr, 1967; Fahn et al., 1987; Movement Disorder Society Task Force on Rating Scales for Parkinson’s Disease, 2003; Goetz et al., 2007; Martinez-Martin et al., 2013; Bloem et al., 2016).

#### Schwab and England daily living activities scale

Schwab and England Daily Living Activities Scale was used in 23.3% (*n* = 30) of the included studies.

*Construct assessed:* Ability to perform ADL.

*Test description:* The S&E is a single-item self-rated scale created specifically for people with PD. Patients are asked to rate their ability to perform ADL from 0% (bedridden) to 100% (completely independent). This instrument takes about 5 min to administer (Schwab and England, 1968; Siderowf, 2010).

*Clinimetric properties:* The test-retest and inter-rater reliabilities of the instrument are both adequate (ICC = 0.7 and 0.6, respectively). A 10-point change was reported as the minimum clinically significant difference (Schwab and England, 1968: Ramaker et al., 2002; Siderowf, 2010; Dal Bello-Haas et al., 2011).

*Feasibility:* Based on patients’ own perceptions, the S&E provides a global impression of patients’ level of independence in performing ADL. Because it is a single-item scale, it is very quick and easy to use, and no prior training is required. It is impossible to differentiate between the most difficult activities and precise limitations (Ramaker et al., 2002; Siderowf, 2010).

#### Movement disorder society-unified Parkinson’s disease rating scale

Movement Disorder Society-Unified Parkinson’s Disease Rating Scale Part II was used in 7.0% (*n* = 9) of the included studies.

*Construct assessed:* Disease severity.

*Test description:* The MDS-UPDRS preserves the UPDRS’ four-part structure, with 65 items and five possible answers: 0 = normal, 1 = slight, 2 = mild, 3 = moderate, and 4 = severe. Parts I (items 1.7–1.13) and II have been designed to fit into a patient/caregiver questionnaire format, allowing them to be completed without the assistance of the investigator. All questions in Part I that deal with complex behaviors (items 1.1–1.6) and all questions in Part IV that deal with motor fluctuations and dyskinesias require the investigator to conduct the interview. Part III keeps the objective assessments of parkinsonism but has now more detailed instructions (Martinez-Martin et al., 2013).

*Clinimetric properties:* The MDS-UPDRS, which was created specifically for people with PD, has high internal consistency (Cronbach’s alpha = 0.79–0.93 across parts) and is highly correlated with the original UPDRS (*q* = 0.96). It also has an excellent construct validity (*r* = 0.92). The instrument has strong concurrent validity based on high correlations with the UPDRS (total score *r* = 0.96), as well as between the individual parts of the two scales. According to Rodriguez-Blazquez et al. (2013), the part II of MDS-UPDRS correlates with other disability measures (Rapid Assessment of Disability Scale and Clinical Impression of Severity Index for PD, *r* = 0.70–0.80) and quality of life scales (Parkinson’s Disease Questionnaire-8 and EQ-5D, *r* = −0.46 to 0.74), which proves useful for assessing disability in PD (Forjaz and Martinez-Martin, 2006; Martinez-Martin et al., 2013; Rodriguez-Blazquez et al., 2013).

*Feasibility:* The MDS-UPDRS has the advantage of being a more complete instrument, with better instructions for quoting the different items. However, as the number of questions has increased, it will take longer to complete the full scale. The assessment of ADL is performed through Part II, which, in addition to being validated to be used independently, is built in a self-completed questionnaire format. It can be completed by the patient and/or the caregiver.

#### The barthel activities of daily living index

The Barthel Index was used in 7.0% (*n* = 9) of the included studies.

*Construct assessed:* Performance in ADL.

*Test description:* The Barthel Index was designed to monitor progression in individuals with chronic diseases who are undergoing rehabilitation. It is a 10-item ordinary scale that assesses a person’s ability to conduct ADLs independently, such as feeding, bathing, grooming, dressing, bladder control, bowel control, toilet use, transfers (bed to chair and back), mobility (on a level surface), and stairs (ascending and descending). It can take 5–20 min depending on how the assessment is performed (self-report vs. direct observation). Higher scores imply greater functional independence, with a severity cut-off of 0–20 indicating “complete” dependency, 21–60 indicating “severe” dependency, 61–90 indicating “moderate” dependency, and 91–99 indicating “slight” dependency (Morley et al., 2012; Taghizadeh et al., 2020).

*Clinimetric properties:* Although not specifically designed, it has been validated for the PD population. In both medication phases it has an excellent test/retest reliability (ICC = 0.84 in ON, ICC = 0.77 in OFF), inter-rater/intra-rater reliability (ICC = 0.91 in ON, ICC = 0.90 in OFF) and internal consistency (Cronbach’s alpha = 0.85 in ON, Cronbach’s alpha = 0.88 in OFF). It also has moderate to high convergent validity in ON and OFF phases (ρ = 0.48–0.82) (Collin et al., 1988; Wade and Collin, 1988; Morley et al., 2012; Taghizadeh et al., 2020).

*Feasibility:* The Barthel Index is a straightforward tool that takes only a few minutes to complete and provides a global but comprehensive view of the patient’s ADL performance. It can be used in both the early and advanced stages of the disease because it can be used as a self-completion questionnaire or for direct observation. The rating system uses the numbers 0–unable, 1–requires assistance, and 2–independent, which does not allow for a detailed description of the difficulties in each task (Wade and Collin, 1988).

#### Lawton-brody instrumental activities of daily living scale

The Lawton-Brody scale was used in 4.7% (*n* = 6) of the included studies.

*Construct assessed:* Independent living skills.

*Test description:* Lawton and Brody developed this scale in 1969 to measure disability levels and assess parameters in community-dwelling older adults. This scale includes eight items, including telephone use, shopping, food preparation, housekeeping, laundry, public transportation use, self-medication management, and financial management. Patients are graded on a scale of 0 (cannot or partially perform) to 1 (can perform) based on their highest level of functioning. The total score ranges from 0 (low functioning, dependent) to 8 (high functioning, independent). The assessment takes about 10–15 min to complete (Graf, 2008).

*Clinimetric properties:* The clinimetric properties of the scale have not been assessed in the PD population. In older adults the instrument has adequate inter-rater reliability (ICC = 0.85). Its validity was tested by determining the correlation with four scales that measure domains of functional status: Physical Classification (6-point rating of physical health), Mental Status Questionnaire (10-point test of orientation and memory), Behavior and Adjustment rating scales (4–6-point measure of intellectual, person, behavioral, and social adjustment), and the Physical Self-Maintenance Scale (6-item ADLs) and all the correlations were significant at the.01 or.05 levels. The scale has also been validated for people with dementia, showing a moderate internal consistency and excellent test-retest reliability (Edwards, 1990; Graf, 2008; Hassani Mehraban et al., 2014; The Memora Group et al., 2021).

*Feasibility:* Unlike the most, the Lawton-Brody scale is focused on instrumental ADL. Despite being an easy and quick to use instrument, it may not be sensitive to small changes. Since some items, such as meal preparation and laundry, are the tasks and responsibilities not performed by institutionalized patients (and men in some cultures) its use is not advised, due to the fact that the lack of responsiveness to some questions is due to cultural differences, not because of the inability to perform those tasks.

#### Functional independence measure

The Functional Independence Measure (FIM) was used in 3.9% (*n* = 5) of the included studies.

*Construct assessed:* Disability.

*Test description:* The FIM is a seven-level ordinal scale with 18 items that assess a patient’s level of disability as well as changes in their status in response to a therapeutic intervention. The domains assessed are self-care independence, such as sphincter control, transfers, locomotion, communication, and social cognition, which are usually assessed through observation. The level of assistance required is used to grade functional status, which ranges from total independence to total assistance. The total score ranges from 18 to 126, with each item having a value ranging from 1 (complete dependence) to 7 (complete independence) (Dodds et al., 1993).

*Clinimetric properties:* The clinimetric properties of the scale have not been assessed in the PD population. Studies in older adults have shown an excellent test-retest reliability (ICC = 0.98) and studies in neurological disorders (not specified which) showed excellent internal consistency (Cronbach’s alpha = 0.95) (Keith et al., 1987; Dodds et al., 1993; Linacre et al., 1994; Hobart et al., 2001; Hsueh, 2002; Coster et al., 2006; Ng et al., 2007; Ellis et al., 2008).

*Feasibility:* The FIM provides realistic and detailed information about a patient’s level of self-care disability. It has the drawbacks of requiring training, being time-consuming (it takes 30–45 min to administer) and requiring environmental conditions that allow self-care tasks to be performed.

#### Alzheimer’s disease cooperative study – activities of daily living inventory

The Alzheimer’s Disease Cooperative Study – Activities of Daily Living Inventory (ADCS-ADL) was used in 3.9% (*n* = 5) of the included studies.

*Construct assessed:* Ability to perform ADLs.

*Test description:* The ADCS-ADL is a questionnaire administered by the rater, in in-person assessments, or by telephone. It was developed to identify which ADLs are useful for the assessment of patients in clinical trials in the Alzheimer’s disease (AD) field. It includes 23 items, 6 basic ADL items, and 17 instrumental ADL items. The total score ranges from 0 to 78, with a lower score indicating greater severity (Galasko et al., 1997).

*Clinimetric properties: The* ADCS-ADL was constructed specifically for use with AD patients. The clinimetric properties of the scale have not been assessed in the PD population (Galasko et al., 1997; Kahle-Wrobleski et al., 2014).

*Feasibility:* It is a time-consuming instrument, based on the patient/caregivers’ perspective on the ability to perform ADL. It was developed for AD and research purposes.

## Discussion

According to our review, ADL was used as an outcome in 129 studies, with 114 (88.4%) using it as the primary outcome. A total of 37 different measurement instruments were used. Of the eight most used measurement instruments, the majority focused on the assessment of basic ADL, with only three including both basic and instrumental ADL assessment.

### Recommended measurement instruments

According to our results, two measurement instruments can be classified as recommended: the MDS-UPDRS and the Schwab & England ADL scale (S&E scale). In some of the included studies, these scales were combined. Only the MDS-UPDRS assesses basic and instrumental ADL.

Regarding the MDS-UPDRS, both the full scale and the independent use of the section dedicated to ADL (Part II), proved to be useful in measuring ADL. The full scale can provide a more comprehensive perspective on how disease severity limits patients’ ability to perform ADL since, besides the patient questionnaire focused on ADL, it includes a motor assessment (a more objective measure) and a questionnaire about non-motor aspects and motor complications associated with PD. It also specifies if a patient was in an ON- or OFF-medication state. However, this is a time-consuming scale, for which the independent use of Part II can be, in some circumstances, a more feasible measurement instrument to evaluate ADL in PD patients.

While the MDS-UPDRS Part II covers both specific basic and instrumental ADL and explores the difficulty presented by the patient in performing each task, the S&E scale provides a global perspective of the level of independence in performing tasks in general. This can be useful in circumstances where a quick and global perspective of a patient’s level of disability is needed.

Although UPDRS complies with all the criteria required to achieve the level of “recommended” and was the most widely used scale for the clinical study of PD for several years, it was replaced by the MDS-UPDRS due to the need to correct some flaws and ambiguities present in the scale and include the evaluation of some relevant missing items. We propose that the MDS recommendation of using the revised version of the scale (the MDS-UPDRS) be followed in order to reduce the variability of the instruments used to assess ADL.

### Suggested measurement instruments

Of the eight most used measurement instruments, only one, the Barthel Index, achieved the level of suggested. This is an instrument focused on basic ADL.

The Barthel Index provides a more complete perspective of patients’ ability to perform ADL than the S&E scale, but a less detailed perspective when compared with the MDS-UPDRS Part II. This measurement instrument was not specifically developed for PD patients, therefore, it does not have a disease-specific perspective when assessing ADL. It is a scale that can be used to evaluate patients through observation but is most often used as a self-completion questionnaire, which can be completed by the patient or caregiver. It has the advantage of being a scale that is simple to use and capable of being filled in by third parties in cases of cognitive deficit (common in advanced stages of the disease). It has the disadvantage of only covering basic ADL and not instrumental ADL. Future studies should study the clinimetric properties of the scale in people with PD. It is a very useful and feasible non-disease-specific instrument to assess ADL for example in institutionalized patients or rehabilitation centers.

### Listed measurement instruments

Three of the eight most used measurement instruments were classified as listed: the Lawton-Brody scale, the FIM, and the ADCS-ADL. Clinimetric properties in the PD population are not available for any of these instruments. Only the ADCS-ADL assesses basic and instrumental ADL, the Lawton-Brody scale is the only instrument (of the eight analyzed) that only assesses instrumental ADL.

The Lawton-Brody scale has the added value of approaching instrumental ADL. However, it does not include the assessment of basic ADL and is only applicable in independent, non-institutionalized/hospitalized patients. Its use may be conditioned in some cultures where men do not perform certain tasks related to housekeeping (e.g., laundry). As PD is more common in men and is also a progressive disease, these factors may limit the use of the scale in clinical practice and research.

The FIM addresses basic ADL by dividing them into motor (Self-care, Sphincter control, Transfers, Locomotion) and cognitive (Communication, Social cognition). ADL can be assessed through observation or interview. Although it takes between 35 and 40 min to administer, it is a frequently used measurement instrument in health institutions for patients with different diagnoses, since it allows the monitoring of the patient’s evolution in response to both disease progression and therapeutic interventions, it is not limited to any stage of the disease and provides a multidisciplinary perspective.

The use of the ADCS-ADL, an instrument developed for patients with AD and not validated in PD, may, at first glance come as a surprise. In our opinion, the fact that this instrument is one of the most used is related to the fact that it evaluates, in a detailed way, an extensive set of ADL, including basic and instrumental ADL. Excluding the MDS-UPDRS, the remaining instruments analyzed here only assess one type of ADL, and the MDS-UPDRS itself does not present such a complete assessment. For research purposes, the ADCS-ADL could be a useful instrument, if its clinimetric properties are studied in PD. Its use in clinical practice is limited by the time it takes to apply.

### Other potential useful measurement instruments

One of the included studies used wearable sensors to evaluate ADL.

The traditional measurement instruments, while accessible and useful, have certain limitations. These include: (1) only providing brief “snapshots” of a patient’s performance, with limited ability to capture how it fluctuates through the day and over time); (2) being subject to intra- and inter-rater variability; (3) requiring an in-person at the very least a telephone assessment; and (4) being time-consuming.

To overcome the limitations of traditional clinical scales, a plethora of new and improved devices have been developed thanks to technological advancements. These have the added value of: (1) capturing the full complexity and diversity of PD symptoms with higher sensitivity and accuracy; (2) providing a more realistic portrayal of patients’ performance; and (3) enabling closer monitoring of therapy response. However, while technology-based objective measures (TOMs) are very promising, some aspects of them need to be improved. The main disadvantage is that, despite significant and rapid advancements in device characteristics, algorithm development has lagged. Currently, issues like algorithmically analyzing captured data, defining truly relevant clinical information, and displaying it synthetically and intuitively need to be addressed.

Future research should explore the use of wearable sensors to monitor ADL in patients with PD, preferably in free-living and passively.

## Conclusion

From the eight most used measurement instruments, two are classified as recommended: the MDS-UPDRS and the S&E scale. The full MDS-UPDRS can provide a more comprehensive perspective of patients’ ability to perform ADL, however, it is time-consuming to use in clinical routine. To overcome this difficulty, the MDS-UPDRS Part II, which focuses on ADL and assesses it through a self-administered questionnaire, can be used as an independent measurement instrument. If a more global perspective of patients’ independence in performing ADLs is sought, the S&E scale is a better option. As the development of TOMs continues, we believe that they might become the best tool to assess ADL in a real, unsupervised context.

## Data availability statement

The original contributions presented in this study are included in the article/supplementary material, further inquiries can be directed to the corresponding author.

## Author contributions

JF and RB-M contributed to the conception of the study. RB-M, CR, and DB conducted the literature search and study selection. AF and RB-M performed data extraction and data analysis and drafted the manuscript. All authors contributed to manuscript revision, read, and approved the submitted version.

## Conflict of interest

The authors declare that the research was conducted in the absence of any commercial or financial relationships that could be construed as a potential conflict of interest.

## Publisher’s note

All claims expressed in this article are solely those of the authors and do not necessarily represent those of their affiliated organizations, or those of the publisher, the editors and the reviewers. Any product that may be evaluated in this article, or claim that may be made by its manufacturer, is not guaranteed or endorsed by the publisher.

## References

[B1] Antonini A. Martinez-Martin P. Chaudhuri R. K. Merello M. Hauser R. Katzenschlager R. (2011). Wearing-off scales in Parkinson’s disease: critique and recommendations: scales to assess wearing-off in PD. *Mov. Disord.* 26 2169–2175. 10.1002/mds.23875 21780180

[B2] Bloem B. R. Marinus J. Almeida Q. Dibble L. Nieuwboer A. Post B. (2016). Measurement instruments to assess posture, gait, and balance in Parkinson’s disease: critique and recommendations. *Mov. Disord.* 31 1342–1355. 10.1002/mds.26572 26945525

[B3] Bloem B. R. Okun M. S. Klein C. (2021). Parkinson’s disease. *Lancet* 397 2284–2303. 10.1016/S0140-6736(21)00218-X 33848468

[B4] Collin C. Wade D. T. Davies S. Horne V. (1988). The barthel ADL index: a reliability study. *Int. Disabil. Stud.* 10 61–63. 10.3109/09638288809164103 3403500

[B5] Coster W. Haley S. Jette A. (2006). Measuring patient-reported outcomes after discharge from inpatient rehabilitation settings1. *J. Rehabil. Med.* 38 237–242. 10.1080/16501970600609774 16801206

[B6] Dal Bello-Haas V. Klassen L. Sheppard M. S. Metcalfe A. (2011). Psychometric properties of activity, self-efficacy, and quality-of-life measures in individuals with parkinson disease. *Physiother. Can.* 63 47–57. 10.3138/ptc.2009-08 22210979 PMC3024195

[B7] De Vriendt P. Gorus E. Cornelis E. Velghe A. Petrovic M. Mets T. (2012). The process of decline in advanced activities of daily living: a qualitative explorative study in mild cognitive impairment. *Int. Psychogeriatr.* 24 974–986. 10.1017/S1041610211002766 22301014

[B8] Dodds T. A. Martin D. P. Stolov W. C. Deyo R. A. (1993). A validation of the functional independence measurement and its performance among rehabilitation inpatients. *Arch. Phys. Med. Rehabil.* 74 531–536. 10.1016/0003-9993(93)90119-U 8489365

[B9] Edemekong P. F. Bomgaars D. L. Sukumaran S. Levy S. B. (eds) (2022). “Activities of daily living,” in *StatPearls*, (Treasure Island, FL: StatPearls Publishing). 29261878

[B10] Edwards M. M. (1990). The reliability and validity of self-report activities of daily living scales. *Can. J. Occup. Ther.* 57 273–278. 10.1177/000841749005700507

[B11] Elble R. Bain P. João Forjaz M. Haubenberger D. Testa C. Goetz C. G. (2013). Task force report: scales for screening and evaluating tremor: critique and recommendations: tremor scales. *Mov. Disord.* 28 1793–1800. 10.1002/mds.25648 24038576

[B12] Ellis T. Katz D. I. White D. K. DePiero T. J. Hohler A. D. Saint-Hilaire M. (2008). Effectiveness of an inpatient multidisciplinary rehabilitation program for people with parkinson disease. *Phys. Ther.* 88 812–819. 10.2522/ptj.20070265 18436568

[B13] Fahn S. Marsden C. Goldstein M. Calne D. (1987). Recent developments in Parkinson’s disease. *Macmillan Healthc. Inform.* 153–63 293–304.

[B14] Forjaz M. J. Martinez-Martin P. (2006). Metric attributes of the unified Parkinson’s disease rating scale 3.0 battery: part II, construct and content validity: validity. *Mov. Disord.* 21 1892–1898. 10.1002/mds.21071 16958134

[B15] Foster E. R. (2014). Instrumental activities of daily living performance among people with Parkinson’s disease without dementia. *Am. J. Occup. Ther.* 68 353–362. 10.5014/ajot.2014.010330 24797199 PMC4011459

[B16] Galasko D. Bennett D. Sano M. Ernesto C. Thomas R. Grundman M. (1997). An inventory to assess activities of daily living for clinical trials in Alzheimer’s disease. The Alzheimer’s Disease Cooperative Study. *Alzheimer Dis. Assoc. Disord.* 11 (Suppl. 2), S33–S39. 10.1097/00002093-199700112-00005 9236950

[B17] Goetz C. G. Fahn S. Martinez-Martin P. Poewe W. Sampaio C. Stebbins G. T. (2007). Movement disorder society-sponsored revision of the unified Parkinson’s disease rating scale (MDS-UPDRS): process, format, and clinimetric testing plan. *Mov. Disord.* 22 41–47. 10.1002/mds.21198 17115387

[B18] Graf C. (2008). The lawton instrumental activities of daily living (IADL) Scale. *Am. J. Nurs.* 108 52–62. 10.1097/01.NAJ.0000314810.46029.74 18367931

[B19] Hagell P. (2019). Measuring activities of daily living in Parkinson’s disease: on a road to nowhere and back again? *Measurement* 132 109–124. 10.1016/j.measurement.2018.09.050

[B20] Hariz G. M. Forsgren L. (2011). Activities of daily living and quality of life in persons with newly diagnosed Parkinson’s disease according to subtype of disease, and in comparison to healthy controls: ADL in early Parkinson’s disease. *Acta Neurol. Scand.* 123 20–27. 10.1111/j.1600-0404.2010.01344.x 20199514

[B21] Hariz G. M. Lindberg M. Hariz M. I. Bergenheim A. T. (2003). Does the ADL part of the unified Parkinson’s disease rating scale measure ADL? An evaluation in patients after pallidotomy and thalamic deep brain stimulation. *Mov. Disord.* 18 373–381. 10.1002/mds.10386 12671942

[B22] Hassani Mehraban A. Soltanmohamadi Y. Akbarfahimi M. Taghizadeh G. (2014). Validity and reliability of the persian version of lawton instrumental activities of daily living scale in patients with dementia. *Med. J. Islam Repub. Iran* 28:25. 25250267 PMC4153527

[B23] Hobart J. C. Lamping D. L. Freeman J. A. Langdon D. W. McLellan D. L. Greenwood R. J. (2001). Evidence-based measurement: which disability scale for neurologic rehabilitation? *Neurology* 57 639–644. 10.1212/WNL.57.4.639 11524472

[B24] Hoehn M. M. Yahr M. D. (1967). Parkinsonism: onset, progression, and mortality. *Neurology* 17 427–427. 10.1212/WNL.17.5.427 6067254

[B25] Hsueh I. P. (2002). Comparison of the psychometric characteristics of the functional independence measure, 5 item Barthel index, and 10 item Barthel index in patients with stroke. *J. Neurol. Neurosurg. Psychiatry* 73 188–190. 10.1136/jnnp.73.2.188 12122181 PMC1737984

[B26] Kahle-Wrobleski K. Coley N. Lepage B. Cantet C. Vellas B. Andrieu S. (2014). Understanding the complexities of functional ability in Alzheimer’s disease: more than just basic and instrumental factors. *Curr. Alzheimer Res.* 11 357–366. 10.2174/1567205011666140317101419 24635843 PMC4021450

[B27] Katz S. (1983). Assessing self-maintenance: activities of daily living, mobility, and instrumental activities of daily living. *J. Am. Geriatr. Soc.* 31 721–727. 10.1111/j.1532-5415.1983.tb03391.x 6418786

[B28] Keith R. A. Granger C. V. Hamilton B. B. Sherwin F. S. (1987). The functional independence measure: a new tool for rehabilitation. *Adv. Clin. Rehabil.* 1 6–18. 3503663

[B29] Lee S. Y. Kim S. K. Cheon S. M. Seo J. W. Kim M. A. Kim J. W. (2016). Activities of daily living questionnaire from patients’ perspectives in Parkinson’s disease: a cross-sectional study. *BMC Neurol.* 16:73. 27206611 10.1186/s12883-016-0600-9 PMC4875616

[B30] Linacre J. M. Heinemann A. W. Wright B. D. Granger C. V. Hamilton B. B. (1994). The structure and stability of the functional independence measure. *Arch. Phys. Med. Rehabil.* 75 127–132. 10.1016/0003-9993(94)90384-0 8311667

[B31] Liu Z. Heffernan C. Tan J. (2020). Caregiver burden: a concept analysis. *Int. J. Nurs. Sci.* 7 438–445. 10.1016/j.ijnss.2020.07.012 33195757 PMC7644552

[B32] Martínez-Martín P. Benito-León J. Alonso F. Catalán M. J. Pondal M. Tobías A. (2003). Patients’, doctors’, and caregivers’ assessment of disability using the UPDRS-ADL section: are these ratings interchangeable: assessment of disability Using the UPDRS. *Mov. Disord.* 18 985–992. 10.1002/mds.10479 14502665

[B33] Martinez-Martin P. Prieto L. Forjaz M. J. (2006). Longitudinal metric properties of disability rating scales for Parkinson’s disease. *Value Health* 9 386–393. 10.1111/j.1524-4733.2006.00131.x 17076869

[B34] Martinez-Martin P. Rodriguez-Blazquez C. Alvarez-Sanchez M. Arakaki T. Bergareche-Yarza A. Chade A. (2013). Expanded and independent validation of the movement disorder society–unified Parkinson’s disease rating scale (MDS-UPDRS). *J. Neurol.* 260 228–236. 10.1007/s00415-012-6624-1 22865238

[B35] Morley D. Selai C. Thompson A. (2012). The self-report Barthel Index: preliminary validation in people with Parkinson’s disease: self-report barthel index. *Eur. J. Neurol.* 19 927–929. 10.1111/j.1468-1331.2011.03592.x 22117585

[B36] Movement Disorder Society Task Force on Rating Scales for Parkinson’s Disease (2003). The unified Parkinson’s disease rating scale (UPDRS): status and recommendations. *Mov. Disord.* 18 738–750. 10.1002/mds.10473 12815652

[B37] Ng Y. S. Jung H. Tay S. S. Bok C. W. Chiong Y. Lim P. A. C. (2007). Results from a prospective acute inpatient rehabilitation database: clinical characteristics and functional outcomes using the functional independence measure. *Ann. Acad, Med. Singap.* 36 3–10. 17285180

[B38] Nguyen H. Lebel K. Bogard S. Goubault E. Boissy P. Duval C. (2018). Using inertial sensors to automatically detect and segment activities of daily living in people with Parkinson’s disease. *IEEE Trans. Neural Syst. Rehabil. Eng.* 26 197–204. 10.1109/TNSRE.2017.2745418 28858808

[B39] Ramaker C. Marinus J. Stiggelbout A. M. van Hilten B. J. (2002). Systematic evaluation of rating scales for impairment and disability in Parkinson’s disease. *Mov. Disord.* 17 867–876. 10.1002/mds.10248 12360535

[B40] Rodriguez-Blazquez C. Rojo-Abuin J. M. Alvarez-Sanchez M. Arakaki T. Bergareche-Yarza A. Chade A. (2013). The MDS-UPDRS Part II (motor experiences of daily living) resulted useful for assessment of disability in Parkinson’s disease. *Parkinson. Relat. Disord.* 19 889–893. 10.1016/j.parkreldis.2013.05.017 23791519

[B41] Schenkman M. Cutson T. M. Zhu C. W. Whetten-Goldstein K. A. (2002). Longitudinal Evaluation of patients’ perceptions of Parkinson’s disease. *Gerontologist* 42 790–798. 10.1093/geront/42.6.790 12451160

[B42] Schwab J. England A. (1968). “Projection technique for evaluating surgery in Parkinson’s disease,” in *Proceedings of the Third Symposium on Parkinson’s Disease*, ed. Aldren Turner J. W. (London: E & S Livingstone), 152–157.

[B43] Siderowf A. (2010). “Schwab and england activities of daily living scale,” in *Encyclopedia of Movement Disorders*, eds Kompoliti K. Verhagen L. (Amsterdam: Elsevier), 99–100. 10.1016/B978-0-12-374105-9.00070-8

[B44] Siderowf A. McDermott M. Kieburtz K. Blindauer K. Plumb S. Shoulson I. (2002). Test-retest reliability of the unified Parkinson’s disease rating scale in patients with early Parkinson’s disease: results from a multicenter clinical trial. *Mov. Disord.* 17 758–763. 10.1002/mds.10011 12210871

[B45] Taghizadeh G. Martinez-Martin P. Meimandi M. Habibi S. A. H. Jamali S. Dehmiyani A. (2020). Barthel Index and modified Rankin Scale: psychometric properties during medication phases in idiopathic Parkinson disease. *Ann. Phys. Rehabil. Med.* 63 500–504. 10.1016/j.rehab.2019.08.006 31816448

[B46] Terwee C. B. Mokkink L. B. Knol D. L. Ostelo R. W. J. G. Bouter L. M. de Vet H. C. W. (2012). Rating the methodological quality in systematic reviews of studies on measurement properties: a scoring system for the COSMIN checklist. *Qual. Life Res.* 21 651–657. 10.1007/s11136-011-9960-1 21732199 PMC3323819

[B47] The Memora Group, Dufournet M. Moutet C. Achi S. Delphin-Combe F. Krolak-Salmon P. (2021). Proposition of a corrected measure of the Lawton instrumental activities of daily living score. *BMC Geriatr.* 21:39. 10.1186/s12877-020-01995-w 33430781 PMC7802257

[B48] Wade D. T. Collin C. (1988). The Barthel ADL index: a standard measure of physical disability? *Int. Disabil. Stud.* 10 64–67. 10.3109/09638288809164105 3042746

